# 2-Amino-4-*tert*-butyl-5-(2,4-dichloro­benz­yl)thia­zol-3-ium bromide

**DOI:** 10.1107/S1600536810004472

**Published:** 2010-02-10

**Authors:** Jie Du, Jun-Mei Peng, Ling Li, Shi-Hong Cai, Ai-Xi Hu

**Affiliations:** a College of Chemistry and Chemical Engineering, Hunan University, Changsha 410082, People’s Republic of China

## Abstract

The asymmetric unit of the title compound, C_14_H_17_Cl_2_N_2_S^+^·Br^−^, contains one cation and two Br^−^ ions with site symmetry 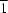. The dihedral angle between the planes of the thia­zol and the dichloro­phenyl rings is 77.8 (6)°. In the crystal, the ions are connected by N–H⋯Br hydrogen bonds.

## Related literature

For background information and related structures, see: Cao *et al.* (2007 ▶); Hu *et al.* (2008 ▶); Marcantonio *et al.* (2002 ▶); Xu *et al.* (2007 ▶).
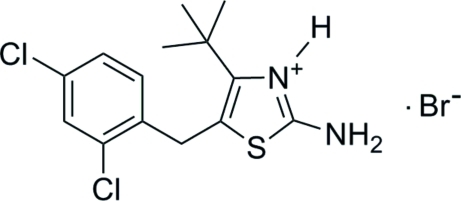


## Experimental

### 

#### Crystal data

C_14_H_17_Cl_2_N_2_S^+^·Br^−^
*M* *_r_* = 396.17
Triclinic, 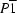

*a* = 8.7797 (5) Å
*b* = 9.3898 (5) Å
*c* = 11.8430 (7) Å
α = 103.960 (1)°
β = 91.102 (1)°
γ = 116.648 (1)°
*V* = 837.66 (8) Å^3^
*Z* = 2
Mo *K*α radiation
μ = 2.89 mm^−1^
*T* = 173 K
0.43 × 0.31 × 0.22 mm

#### Data collection

Bruker SMART 1000 CCD diffractometer
Absorption correction: multi-scan (*SADABS*; Sheldrick, 2004 ▶) *T*_min_ = 0.370, *T*_max_ = 0.569
6572 measured reflections
3255 independent reflections
2726 reflections with *I* > 2σ(*I*)
*R*_int_ = 0.021

#### Refinement

*R*[*F* ^2^ > 2σ(*F* ^2^)] = 0.029
*wR*(*F* ^2^) = 0.099
*S* = 1.08
3255 reflections
187 parameters
H-atom parameters constrained
Δρ_max_ = 0.37 e Å^−3^
Δρ_min_ = −0.33 e Å^−3^

### 

Data collection: *SMART* (Bruker, 2001 ▶); cell refinement: *SAINT-Plus* (Bruker, 2003 ▶); data reduction: *SAINT-Plus*; program(s) used to solve structure: *SHELXTL* (Sheldrick, 2008 ▶); program(s) used to refine structure: *SHELXTL*; molecular graphics: *SHELXTL*; software used to prepare material for publication: *SHELXTL*.

## Supplementary Material

Crystal structure: contains datablocks I, New_Global_Publ_Block. DOI: 10.1107/S1600536810004472/bt5190sup1.cif

Structure factors: contains datablocks I. DOI: 10.1107/S1600536810004472/bt5190Isup2.hkl

Additional supplementary materials:  crystallographic information; 3D view; checkCIF report

## Figures and Tables

**Table 1:** Hydrogen-bond geometry (Å, °)

*D*—H⋯*A*	*D*—H	H⋯*A*	*D*⋯*A*	*D*—H⋯*A*
N2—H2*B*⋯Br2	0.88	2.48	3.296 (2)	154
N1—H1⋯Br1^i^	0.88	2.47	3.286 (2)	153.7

Symmetry code: (i) 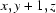.

**Fig. 1.**
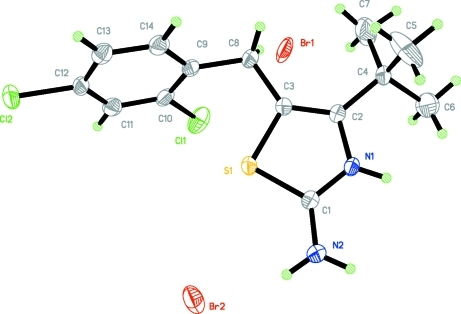
The asymmetric unit of the title compound with atom labels and 50% probability displacement ellipsiods (arbitrary spheres for H atoms).

## supplementary crystallographic information

### Comment

Thiazol compounds have a wide range of biological activity. The title compund was obtained by the reaction of thiurea and 2-bromo-1-(2,4-dichlorophenyl)-4,4-dimethyl-3-pentanone.

### Experimental

A solution with 0.05 mol of thiurea and 0.05 mol of 2-bromo-1-(2,4-dichlorophenyl)-4,4-dimethyl-3-pentanone in 100 ml of ethanol was refluxed, monitoring by TLC (yield 99.5 %; m.p. 507.6–508.5 K). Crystals   were obtained by slow evaporation of an ethanol solution at room temperature.

### Refinement

All H atoms were refined using a riding model, with N—H distances of 0.88 and C—H distances ranging from 0.95 to 0.98 Å, and with *U*_iso_(H) = 1.2U_eq_(C,N) or *U*_iso_(H) = 1.5U_eq_(C_methyl_).

### Crystal data

**Table:**

C_14_H_17_Cl_2_N_2_S^+^·Br^−^	*Z* = 2
*M**_r_* = 396.17	*F*(000) = 400
Triclinic, *P*1	*D*_x_ = 1.571 Mg m^−^^3^
Hall symbol: -P 1	Melting point: 508 K
*a* = 8.7797 (5) Å	Mo *K*α radiation, λ = 0.71073 Å
*b* = 9.3898 (5) Å	Cell parameters from 3934 reflections
*c* = 11.8430 (7) Å	θ = 2.5–27.0°
α = 103.960 (1)°	µ = 2.89 mm^−^^1^
β = 91.102 (1)°	*T* = 173 K
γ = 116.648 (1)°	Block, colorless
*V* = 837.66 (8) Å^3^	0.43 × 0.31 × 0.22 mm

### Data collection

**Table:**

Bruker SMART 1000 CCD diffractometer	3255 independent reflections
Radiation source: fine-focus sealed tube	2726 reflections with *I* > 2σ(*I*)
graphite	*R*_int_ = 0.021
ω scans	θ_max_ = 26.0°, θ_min_ = 1.8°
Absorption correction: multi-scan (*SADABS*; Sheldrick, 2004)	*h* = −10→10
*T*_min_ = 0.370, *T*_max_ = 0.569	*k* = −11→11
6572 measured reflections	*l* = −14→14

### Refinement

**Table:**

Refinement on *F*^2^	Primary atom site location: structure-invariant direct methods
Least-squares matrix: full	Secondary atom site location: difference Fourier map
*R*[*F*^2^ > 2σ(*F*^2^)] = 0.029	Hydrogen site location: inferred from neighbouring sites
*wR*(*F*^2^) = 0.099	H-atom parameters constrained
*S* = 1.08	*w* = 1/[σ^2^(*F*_o_^2^) + (0.0512*P*)^2^ + 0.723*P*] where *P* = (*F*_o_^2^ + 2*F*_c_^2^)/3
3255 reflections	(Δ/σ)_max_ < 0.001
187 parameters	Δρ_max_ = 0.37 e Å^−^^3^
0 restraints	Δρ_min_ = −0.33 e Å^−^^3^

### Special details

**Table:**

Experimental. ^1^H NMR (CDCl_3_,400 MHz) of 4-*tert*-butyl-5-(2,4-dichlorobenzyl)thiazol-2-amine: δ (p.p.m.) 1.30(s, 9H, 3×CH_3_), 4.15(s, 2H, CH_2_),4.83(br, 2H, NH_2_),7.08(d, *J* = 11.2 Hz, 1H, C_6_H_3_ 6-H),7.18(d, *J* = 11.2 Hz, 1H, C_6_H_3_ 5-H),7.38(s, 1H, C_6_H_3_ 3-H).
Geometry. All esds (except the esd in the dihedral angle between two l.s. planes) are estimated using the full covariance matrix. The cell esds are taken into account individually in the estimation of esds in distances, angles and torsion angles; correlations between esds in cell parameters are only used when they are defined by crystal symmetry. An approximate (isotropic) treatment of cell esds is used for estimating esds involving l.s. planes.
Refinement. Refinement of *F*^2^ against ALL reflections. The weighted *R*-factor wR and goodness of fit *S* are based on *F*^2^, conventional *R*-factors *R* are based on *F*, with *F* set to zero for negative *F*^2^. The threshold expression of *F*^2^ > σ(*F*^2^) is used only for calculating *R*-factors(gt) etc. and is not relevant to the choice of reflections for refinement. *R*-factors based on *F*^2^ are statistically about twice as large as those based on *F*, and *R*- factors based on ALL data will be even larger.

### Fractional atomic coordinates and isotropic or equivalent isotropic displacement parameters (Å^2^)

**Table:**

	*x*	*y*	*z*	*U*_iso_*/*U*_eq_	
Br1	0.5000	0.0000	0.5000	0.05632 (19)	
Br2	0.0000	0.5000	0.0000	0.03684 (15)	
Cl1	0.23215 (12)	0.02866 (10)	0.20559 (7)	0.0408 (2)	
Cl2	0.08968 (10)	−0.20337 (10)	−0.26428 (7)	0.0404 (2)	
S1	0.32616 (9)	0.42272 (8)	0.15292 (6)	0.02508 (17)	
C1	0.3491 (3)	0.5970 (3)	0.2559 (2)	0.0225 (6)	
C2	0.5883 (3)	0.5746 (3)	0.3167 (2)	0.0211 (5)	
C3	0.5156 (3)	0.4406 (3)	0.2230 (2)	0.0219 (5)	
C4	0.7575 (4)	0.6530 (3)	0.3986 (2)	0.0265 (6)	
C5	0.8920 (5)	0.7846 (6)	0.3502 (4)	0.0696 (14)	
H5A	0.9056	0.7324	0.2715	0.084*	
H5B	1.0023	0.8389	0.4023	0.084*	
H5C	0.8548	0.8674	0.3456	0.084*	
C6	0.7388 (5)	0.7336 (6)	0.5218 (3)	0.0563 (11)	
H6A	0.7059	0.8197	0.5190	0.084*	
H6B	0.8487	0.7836	0.5736	0.084*	
H6C	0.6497	0.6492	0.5521	0.084*	
C7	0.8196 (5)	0.5284 (5)	0.4073 (4)	0.0635 (13)	
H7A	0.7297	0.4374	0.4318	0.095*	
H7B	0.9241	0.5832	0.4654	0.095*	
H7C	0.8451	0.4841	0.3304	0.095*	
C8	0.5707 (4)	0.3106 (3)	0.1692 (3)	0.0276 (6)	
H8A	0.5796	0.2565	0.2293	0.033*	
H8B	0.6866	0.3664	0.1468	0.033*	
C9	0.4488 (3)	0.1782 (3)	0.0618 (2)	0.0230 (6)	
C10	0.2912 (4)	0.0491 (3)	0.0691 (2)	0.0245 (6)	
C11	0.1777 (4)	−0.0692 (3)	−0.0297 (3)	0.0272 (6)	
H11	0.0703	−0.1556	−0.0229	0.033*	
C12	0.2274 (4)	−0.0561 (3)	−0.1384 (2)	0.0255 (6)	
C13	0.3842 (4)	0.0667 (4)	−0.1501 (3)	0.0266 (6)	
H13	0.4170	0.0712	−0.2257	0.032*	
C14	0.4927 (4)	0.1830 (3)	−0.0495 (3)	0.0271 (6)	
H14	0.6003	0.2686	−0.0570	0.033*	
N1	0.4907 (3)	0.6601 (3)	0.33357 (19)	0.0215 (5)	
H1	0.5211	0.7503	0.3920	0.026*	
N2	0.2419 (3)	0.6618 (3)	0.2595 (2)	0.0304 (6)	
H2A	0.2626	0.7518	0.3155	0.037*	
H2B	0.1501	0.6148	0.2058	0.037*	

### Atomic displacement parameters (Å^2^)

**Table:**

	*U*^11^	*U*^22^	*U*^33^	*U*^12^	*U*^13^	*U*^23^
Br1	0.0824 (4)	0.0488 (3)	0.0407 (3)	0.0505 (3)	−0.0187 (3)	−0.0205 (2)
Br2	0.0325 (2)	0.0267 (2)	0.0393 (3)	0.00678 (19)	−0.01243 (18)	0.00437 (18)
Cl1	0.0576 (5)	0.0352 (4)	0.0299 (4)	0.0215 (4)	0.0178 (4)	0.0092 (3)
Cl2	0.0381 (4)	0.0391 (4)	0.0337 (4)	0.0203 (4)	−0.0137 (3)	−0.0106 (3)
S1	0.0235 (3)	0.0200 (3)	0.0266 (4)	0.0115 (3)	−0.0062 (3)	−0.0042 (3)
C1	0.0233 (13)	0.0222 (13)	0.0209 (13)	0.0118 (11)	0.0017 (10)	0.0022 (11)
C2	0.0217 (13)	0.0184 (13)	0.0209 (13)	0.0094 (11)	0.0019 (10)	0.0018 (10)
C3	0.0195 (13)	0.0192 (13)	0.0236 (13)	0.0087 (11)	0.0002 (10)	0.0009 (11)
C4	0.0242 (14)	0.0234 (14)	0.0251 (14)	0.0111 (12)	−0.0050 (11)	−0.0043 (11)
C5	0.0270 (19)	0.079 (3)	0.074 (3)	−0.003 (2)	−0.0070 (19)	0.030 (3)
C6	0.051 (2)	0.077 (3)	0.0328 (18)	0.040 (2)	−0.0144 (16)	−0.0150 (18)
C7	0.057 (3)	0.049 (2)	0.074 (3)	0.035 (2)	−0.035 (2)	−0.017 (2)
C8	0.0285 (15)	0.0226 (14)	0.0292 (15)	0.0157 (12)	−0.0023 (12)	−0.0041 (12)
C9	0.0257 (14)	0.0197 (13)	0.0266 (14)	0.0162 (12)	−0.0010 (11)	0.0006 (11)
C10	0.0295 (15)	0.0241 (14)	0.0229 (13)	0.0172 (12)	0.0055 (11)	0.0020 (11)
C11	0.0248 (14)	0.0201 (13)	0.0349 (16)	0.0114 (12)	0.0033 (12)	0.0029 (12)
C12	0.0278 (14)	0.0213 (14)	0.0262 (14)	0.0158 (12)	−0.0055 (11)	−0.0035 (11)
C13	0.0348 (16)	0.0282 (15)	0.0239 (14)	0.0201 (13)	0.0045 (12)	0.0080 (12)
C14	0.0259 (15)	0.0211 (14)	0.0327 (15)	0.0112 (12)	0.0056 (12)	0.0043 (12)
N1	0.0241 (11)	0.0183 (11)	0.0201 (11)	0.0115 (10)	−0.0016 (9)	−0.0011 (9)
N2	0.0290 (13)	0.0294 (13)	0.0322 (13)	0.0193 (11)	−0.0043 (10)	−0.0034 (11)

### Geometric parameters (Å, °)

**Table:**

Cl1—C10	1.736 (3)	C7—H7A	0.9800
Cl2—C12	1.742 (3)	C7—H7B	0.9800
S1—C1	1.714 (3)	C7—H7C	0.9800
S1—C3	1.764 (3)	C8—C9	1.516 (4)
C1—N2	1.328 (4)	C8—H8A	0.9900
C1—N1	1.332 (3)	C8—H8B	0.9900
C2—C3	1.342 (4)	C9—C14	1.386 (4)
C2—N1	1.402 (3)	C9—C10	1.394 (4)
C2—C4	1.519 (4)	C10—C11	1.389 (4)
C3—C8	1.515 (4)	C11—C12	1.383 (4)
C4—C7	1.519 (5)	C11—H11	0.9500
C4—C5	1.519 (5)	C12—C13	1.380 (4)
C4—C6	1.522 (4)	C13—C14	1.384 (4)
C5—H5A	0.9800	C13—H13	0.9500
C5—H5B	0.9800	C14—H14	0.9500
C5—H5C	0.9800	N1—H1	0.8800
C6—H6A	0.9800	N2—H2A	0.8800
C6—H6B	0.9800	N2—H2B	0.8800
C6—H6C	0.9800		
			
C1—S1—C3	90.62 (13)	H7A—C7—H7C	109.5
N2—C1—N1	123.9 (2)	H7B—C7—H7C	109.5
N2—C1—S1	125.4 (2)	C3—C8—C9	113.8 (2)
N1—C1—S1	110.71 (19)	C3—C8—H8A	108.8
C3—C2—N1	111.2 (2)	C9—C8—H8A	108.8
C3—C2—C4	132.1 (2)	C3—C8—H8B	108.8
N1—C2—C4	116.6 (2)	C9—C8—H8B	108.8
C2—C3—C8	131.2 (3)	H8A—C8—H8B	107.7
C2—C3—S1	111.4 (2)	C14—C9—C10	117.3 (2)
C8—C3—S1	117.33 (19)	C14—C9—C8	119.8 (3)
C7—C4—C2	112.7 (2)	C10—C9—C8	122.9 (3)
C7—C4—C5	108.5 (3)	C11—C10—C9	122.7 (3)
C2—C4—C5	107.7 (3)	C11—C10—Cl1	117.3 (2)
C7—C4—C6	108.1 (3)	C9—C10—Cl1	120.0 (2)
C2—C4—C6	110.3 (2)	C12—C11—C10	117.3 (3)
C5—C4—C6	109.5 (3)	C12—C11—H11	121.3
C4—C5—H5A	109.5	C10—C11—H11	121.3
C4—C5—H5B	109.5	C13—C12—C11	122.2 (3)
H5A—C5—H5B	109.5	C13—C12—Cl2	119.1 (2)
C4—C5—H5C	109.5	C11—C12—Cl2	118.7 (2)
H5A—C5—H5C	109.5	C12—C13—C14	118.6 (3)
H5B—C5—H5C	109.5	C12—C13—H13	120.7
C4—C6—H6A	109.5	C14—C13—H13	120.7
C4—C6—H6B	109.5	C13—C14—C9	121.9 (3)
H6A—C6—H6B	109.5	C13—C14—H14	119.1
C4—C6—H6C	109.5	C9—C14—H14	119.1
H6A—C6—H6C	109.5	C1—N1—C2	116.1 (2)
H6B—C6—H6C	109.5	C1—N1—H1	122.0
C4—C7—H7A	109.5	C2—N1—H1	122.0
C4—C7—H7B	109.5	C1—N2—H2A	120.0
H7A—C7—H7B	109.5	C1—N2—H2B	120.0
C4—C7—H7C	109.5	H2A—N2—H2B	120.0
			
C3—S1—C1—N2	−179.0 (3)	C14—C9—C10—C11	−1.8 (4)
C3—S1—C1—N1	0.8 (2)	C8—C9—C10—C11	178.1 (2)
N1—C2—C3—C8	179.8 (3)	C14—C9—C10—Cl1	176.7 (2)
C4—C2—C3—C8	4.9 (5)	C8—C9—C10—Cl1	−3.4 (4)
N1—C2—C3—S1	1.3 (3)	C9—C10—C11—C12	0.7 (4)
C4—C2—C3—S1	−173.7 (2)	Cl1—C10—C11—C12	−177.9 (2)
C1—S1—C3—C2	−1.2 (2)	C10—C11—C12—C13	1.2 (4)
C1—S1—C3—C8	180.0 (2)	C10—C11—C12—Cl2	179.3 (2)
C3—C2—C4—C7	−27.2 (5)	C11—C12—C13—C14	−1.9 (4)
N1—C2—C4—C7	158.0 (3)	Cl2—C12—C13—C14	−179.9 (2)
C3—C2—C4—C5	92.4 (4)	C12—C13—C14—C9	0.7 (4)
N1—C2—C4—C5	−82.3 (3)	C10—C9—C14—C13	1.1 (4)
C3—C2—C4—C6	−148.1 (3)	C8—C9—C14—C13	−178.8 (3)
N1—C2—C4—C6	37.1 (4)	N2—C1—N1—C2	179.6 (3)
C2—C3—C8—C9	180.0 (3)	S1—C1—N1—C2	−0.3 (3)
S1—C3—C8—C9	−1.5 (3)	C3—C2—N1—C1	−0.6 (3)
C3—C8—C9—C14	103.4 (3)	C4—C2—N1—C1	175.2 (2)
C3—C8—C9—C10	−76.5 (3)		

### Hydrogen-bond geometry (Å, °)

**Table:**

*D*—H···*A*	*D*—H	H···*A*	*D*···*A*	*D*—H···*A*
N2—H2B···Br2	0.88	2.48	3.296 (2)	154
N1—H1···Br1^i^	0.88	2.47	3.286 (2)	153.7

Symmetry codes: (i) *x*, *y*+1, *z*.
